# Projection of Future Mortality Due to Temperature and Population Changes under Representative Concentration Pathways and Shared Socioeconomic Pathways

**DOI:** 10.3390/ijerph15040822

**Published:** 2018-04-21

**Authors:** Jae Young Lee, Ejin Kim, Woo-Seop Lee, Yeora Chae, Ho Kim

**Affiliations:** 1 Institute of Health and Environment and Graduate School of Public Health, Seoul National University, 1, Gwanak-ro, Gwanak-gu, Seoul 08826, Korea; jaeyoung.lee@alumni.stanford.edu (J.Y.L.); platin@snu.ac.kr (E.K.); 2 Climate Research Department, APEC Climate Center, 12, Centum 7-ro, Haeundae-gu, Busan 48058, Korea; wslee@apcc21.org; 3 Korea Environment Institute, 370 Sicheong-daero, Sejong 30147, Korea; yrchae@kei.re.kr

**Keywords:** projection, mortality, shared socioeconomic pathways, climate change, global warming, paris agreement

## Abstract

The Paris Agreement aims to limit the global temperature increase to below 2 °C above pre-industrial levels and to pursue efforts to limit the increase to even below 1.5 °C. Now, it should be asked what benefits are in pursuing these two targets. In this study, we assessed the temperature–mortality relationship using a distributed lag non-linear model in seven major cities of South Korea. Then, we projected future temperature-attributable mortality under different Representative Concentration Pathway (RCP) and Shared Socioeconomic Pathway (SSP) scenarios for those cities. Mortality was projected to increase by 1.53 under the RCP 4.5 (temperature increase by 2.83 °C) and 3.3 under the RCP 8.5 (temperature increase by 5.10 °C) until the 2090s, as compared to baseline (1991–2015) mortality. However, future mortality is expected to increase by less than 1.13 and 1.26 if the 1.5 °C and 2 °C increase targets are met, respectively, under the RCP 4.5. Achieving the more ambitious target of 1.5 °C will reduce mortality by 12%, when compared to the 2 °C target. When we estimated future mortality due to both temperature and population changes, the future mortality was found to be increased by 2.07 and 3.85 for the 1.5 °C and 2 °C temperature increases, respectively, under the RCP 4.5. These increases can be attributed to a growing proportion of elderly population, who is more vulnerable to high temperatures. Meeting the target of 1.5 °C will be particularly beneficial for rapidly aging societies, including South Korea.

## 1. Introduction

The frequent occurrence of high temperature due to global warming has serious health impacts. For example, extreme weather events such as heat waves are seen at increased frequencies, intensities, durations, and spatial extents. These changes are associated with a higher rate of mortality in humans [1,2,3,4,5]. Besides the exposure to elevated temperatures, climate change affects humans in several ways. According to the Intergovernmental Panel on Climate Change (IPCC), the higher the temperature, the greater is the likelihood of droughts, flooding, resource depletion, conflict, and forced migration. Estimating the health burden of climate change is of great interest to researchers, and several studies have examined this burden [6,7,8,9,10,11,12,13,14].

During the 2015 United Nations Climate Change Conference held in Paris, 195 countries of the world strengthened the United Nations Framework Convention on Climate Change by agreeing to limiting the increase in the global average temperature to well below 2 °C above pre-industrial levels and pursuing efforts to limit it to below 1.5 °C [15]. These targets were determined to render the total impact of climate change tolerable. Now, it is important to investigate the health impacts of global temperature increases of 1.5 °C and 2 °C, to determine if such increases are indeed tolerable.

South Korea has one of the fastest aging societies worldwide; therefore, it is an interesting setting to investigate the impact of climate change on human health. This study examined future mortality based on temperature changes under Representative Concentration Pathways (RCPs) as well as based on population changes under Shared Socioeconomic Pathways (SSPs) in seven major cities of South Korea. Under two RCP scenarios, we analyzed the health burden of temperature increases of 1.5 °C and 2 °C to evaluate the importance of these targets in a fast-aging society such as that of South Korea.

## 2. Materials and Methods

### 2.1. Data

The daily non-accidental mortality rates (based on the International Statistical Classification of Diseases and Related Health Problems 10th Revision [ICD-10 codes] A00–R99) as well as the daily maximum temperature and humidity in seven metropolitan cities (Seoul, Busan, Daegu, Incheon, Gwangju, Deajeon, and Ulsan) of South Korea during 1991–2015 were obtained from Statistics Korea and the Korea Meteorological Administration, respectively. The temperature and humidity data were measured in the seven official stations, each located in a different city. The populations of the seven cities in 2015 are summarized in Supplementary Table S1. These seven cities were selected for the study for two reasons. First of all, they were major cities which collectively accounted for about 46% of the total population in South Korea, and, second, the temperatures in these cities were expected to increase more because of the urban heat island effect. Future daily maximum temperatures (until the year 2100), using 25 general circulation models (GCM), were obtained from the Asia-Pacific Economic Cooperation Climate Center located in Busan, South Korea. For these climate models, future maximum temperatures were available under two RCP scenarios (RCP 4.5 and 8.5). The future population estimated under three shared SSPs (SSP 1, 2, and 3) was obtained from the Korea Environment Institute.

### 2.2. Estimation of Temperature Increases above Pre-industrial Levels

According to the Summary for Policymakers (IPCC, 2013), the average global temperature between 1986 and 2005 increased by 0.61 °C above pre-industrial levels. Accordingly, we obtained the temperature increases above pre-industrial levels in seven cities of South Korea by assuming the temperature increase during the same period to be also 0.61 °C. This calculation was expressed as:

(1) $$TI_{y}\;=\;T_{y}-T_{1986-2005}\;+\;0.61\;$$

Here,$\;TI_{y}$ is the 20-year average temperature increase above pre-industrial levels during the 20-year period from y − 9 to y + 10, $T_{y}$ is the 20-year average temperature during the same period, and $T_{1986-2005}$ is the 20-year average temperature between 1986 and 2005.

### 2.3. Association between Temperature and Mortality

We used a distributed lag non-linear model (DLNM) to quantify the excess risk of daily mortality associated with the daily maximum temperature in seven cities of South Korea. The temperature–mortality and –lag-mortality relationships were both modeled using natural cubic spline curves, and the bases of these curves were used to construct a cross basis for the DLNM [16]. Lags were modeled up to 21 days, and a quasi-Poisson distribution was assumed for the distribution of daily mortality. The DLNM is expressed as

(2) $$\log\left[E\left(Y_{t}\right)\right]=CB\left(T_{\max}\right)\;+\;\mathrm{DOW}\;+\;\mathrm{ns}\left(\mathrm{Time},df\;=\;4\;per\;year\right)\;$$

Here, $E\left(Y_{t}\right)$ is the expected number of daily deaths, $CB\left(T_{max}\right)$ is the cross basis of the maximum temperature–lag-mortality relationship, and DOW is the day of the week. The time–mortality relationship is modelled using a natural cubic spline curve with four degrees of freedom per year to control for seasonal and long-term mortality variations.

### 2.4. Temperature-Related Mortality Projection

To project the future temperature-attributable mortality under climate and population changes, we used the equation

(3) $$\begin{array}{cc} MR & =\frac{Future\;daily\;temperature-attributable\;mortality}{Baseline\;daily\;temperature-attributable\;mortality}\;\\ & =\frac{\;\Sigma_{i=1}^{3}M_{b,i}\cdot RR_{i}\cdot POP_{i}}{\Sigma_{i=1}^{3}M_{b,i}}\; \end{array}$$

where $M_{b,i}$ is the baseline daily temperature-attributable mortality for the age group i, $RR_{i}$ is the ratio between the future and baseline temperature-attributable mortality for the age group i, and $POP_{i}$ is the estimated population change for the age group i.

## 3. Results

### 3.1. Maximum Temperature Increases and Population Changes

**Figure 1:**
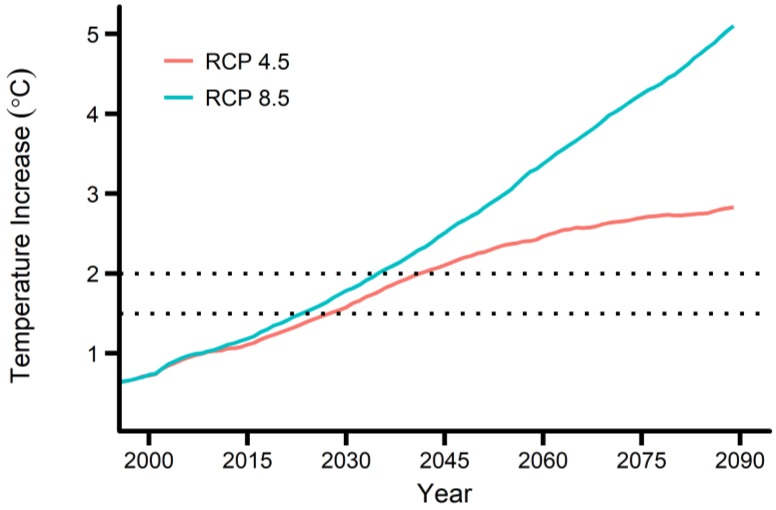
20-year average maximum temperature increases above the pre-industrial baseline for seven major cities of South Korea. RCP, Representative Concentration Pathway.

Figure 1 shows the 20-year average maximum temperature increase above the pre-industrial baseline for the two RCP scenarios. The temperature increase curves were obtained by averaging the future maximum temperatures from 25 GCMs and converting the average to the temperature increase, based on the method in Section 2.2. The 20-year average maximum temperature is expected to increase by 2.83 °C and 5.10 °C until 2089 (averaged during 2080–2099) under the RCP 4.5 and 8.5, respectively. The 1.5 °C and 2 °C increases above the pre-industrial baseline are expected to appear in 2027 (2018–2037) and 2041 (2032–2051) under the RCP 4.5, and in 2023 (2014–2033) and 2035 (2026–2045) under the RCP 8.5.

### 3.2. Population Changes

**Figure 2:**
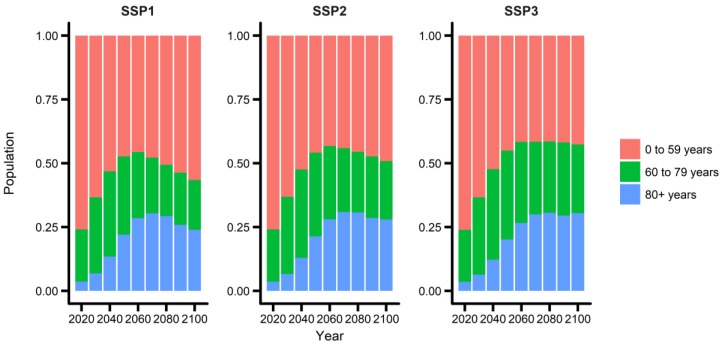
Population composition changes under the SSP 1, 2 and, 3 scenarios in the seven cities. SSP, Shared Socioeconomic Pathway.

Figure 2 shows the predicted population composition changes under the three SSP scenarios (SSP1, 2, and 3). The three SSP scenarios reflect low, intermediate, and high challenges for mitigation and adaptation, respectively. Since South Korea is one of the fastest aging countries worldwide (Lee and Kim, 2016), the portion of the elderly population has been increasing rapidly. The proportion of the “oldest-old” age group (individuals aged ≥80 years) is expected to increase until the 2070s under all three SSP scenarios. However, thereafter, it will slowly decrease under the SSP1 and SSP2 and remain roughly constant under the SSP3. The “oldest-old” age group will account for 25.7%, 28.2%, and 28.9% of the population in the year 2100 under the SSP1, 2, and 3, respectively. Similar findings were observed for the “old” age group (individuals aged ≥60 years); the proportion of this age group is expected to be 45.3%, 51.4%, and 56.3% in the year 2100 under the SSP1, 2, and 3, respectively. The definitions of “oldest-old” (≥80 years) and “old” (≥60 years) populations follow the definitions used in the population ageing report by the United Nations [17]. We observed similar trends for the “all age”, “old age”, and “oldest-old” age groups (Figure S1). Moreover, the aging and population trends were similar across all seven cities (Figure S2).

### 3.3. Mortality Prediction Due to Temperature Changes

**Figure 3:**
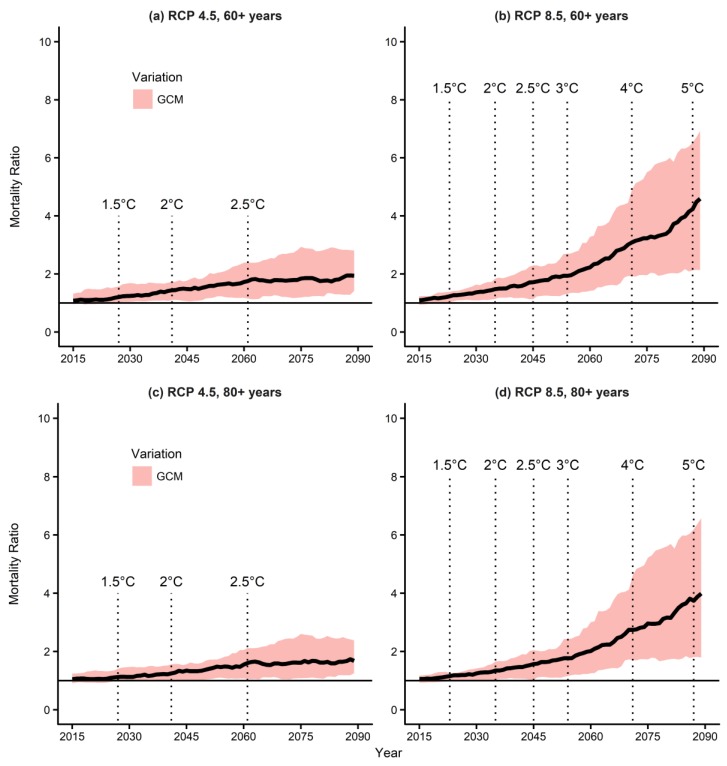
Mortality ratios due to temperature changes under the two RCP scenarios: (**a**) RCP 4.5, individuals aged ≥60 years, (**b**) RCP 8.5, individuals aged ≥60 years, (**c**) RCP 4.5, individuals aged ≥80 years, and (**d**) RCP 8.5, individuals aged ≥80 years. GCM, general circulation model; RCP, Representative Concentration Pathway.

Figure 3 shows the mortality ratios (MR) for the baseline (1991–2015) and future periods according to temperature changes in the “old” (aged ≥60 years) and “oldest-old” (aged ≥80 years) age groups, and Figure S3 shows the same for the total population. The mortality ratios were obtained based on the baseline temperature and mortality relationships shown in Figure S4. We assumed that the baseline population stays constant until 2100. To understand how the Paris Agreement may affect future temperature-related mortality in seven major cities of South Korea, we assessed the effects of temperature increases of 1.5 °C and 2 °C (Figure 3 and Figure S3). The MRs for the 1.5 °C and 2 °C increases were below 1.20 and 1.44 under the RCP 4.5 and below 1.23 and 1.48 under the RCP 8.5, indicating that, if the temperature increase can be limited to below 1.5 °C, mortality can be reduced by 12~13%, when compared to a temperature increase of 2 °C.

Supplementary Table S2 summarizes the MRs according to temperature increases of 1.5 °C, 2 °C, 2.5 °C, 3 °C, 4 °C, and 5 °C. The MRs for 2.5–5 °C were added for reference. We found similar MRs across all three age groups (see Table S2). The MR under the RCP 4.5 increased until the 2060s and saturated at 1.5~1.8; in contrast, the MR under the RCP 8.5 kept increasing and reached 3.3~4.2 in the 2090s.

### 3.4. Mortality Prediction Due to Temperature and Population Changes

**Figure 4:**
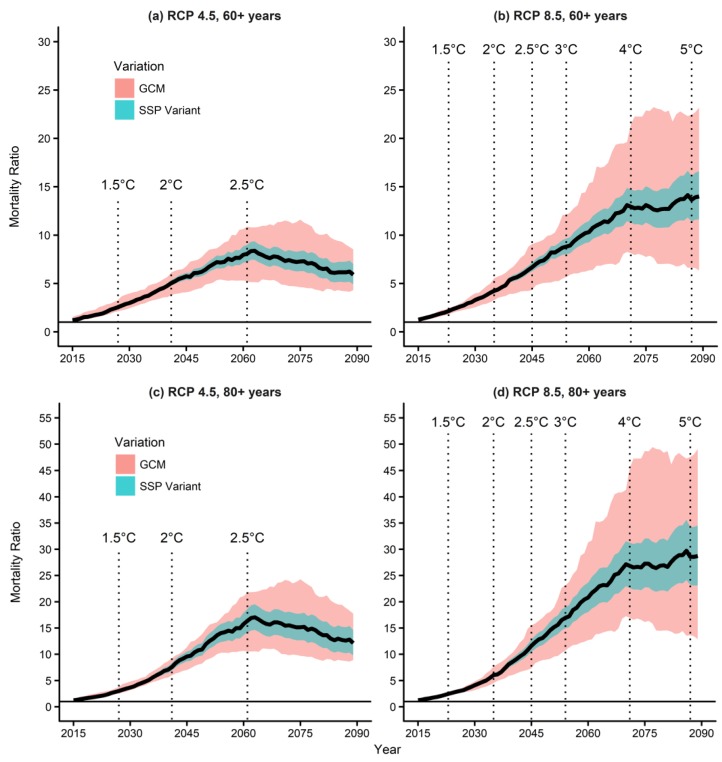
Mortality ratios according to temperature and population changes under different RCP and SSP scenarios: (**a**) RCP 4.5, individuals aged ≥60 years, (**b**) RCP 8.5, individuals aged ≥60 years, (**c**) RCP 4.5, individuals aged ≥80 years, and (**d**) RCP 8.5, individuals aged ≥80 years. GCM, general circulation model; RCP, Representative Concentration Pathway; SSP, shared Socioeconomic Pathway.

When considering both temperature and population changes, future mortality was expected to increase further. The MRs under the RCP 4.5 and SSP2 peaked during the 2060s and reached 6.0, 8.1, and 16.4 in the “all age”, “old age”, and “oldest-old” age groups, respectively, while those under the RCP 8.5 and SSP2 kept increasing until they reached 10.1, 13.7, and 28.6, respectively, during the 2090s (Figure 4 and Figure S5).

The MRs according to temperature and population changes are summarized in Supplementary Table S3. Notably, the values of the MRs were much higher when considering the population changes than the corresponding ones not considering the population changes (see Table S2). Moreover, the MR for the “oldest-old” age group was more than twice higher than that for the other age groups. We did not observe this when only changes in temperature were considered. This is likely because the proportion of the elderly population has been increasing rapidly in South Korea, thereby proportionally increasing mortality in this group.

When considering the population changes, MR also increased for the 1.5 °C and 2 °C temperature increases. In the “old” age group under the RCP 4.5, the future MRs increased up to 2.55 and 5.04, respectively, at the points at which the temperatures increased by 1.5 °C and 2 °C. The MRs increased up to 2.14 and 4.23, respectively, under the RCP 8.5 (Figure 3a,b and Table S3). In the “oldest-old” age group, the MRs reached up to 3.01 and 7.57 under the RCP 4.5 and up to 2.45 and 6.04 under the RCP 8.5 for 1.5 °C and 2 °C increase in temperature, respectively (Figure 3c,d and Table S3). These findings indicate that considering the population changes increases the benefit of limiting the global temperature increase to 1.5 °C, in particular in aging societies such as South Korea.

### 3.5. Mortality Prediction Due to Extreme Temperature Changes

**Figure 5:**
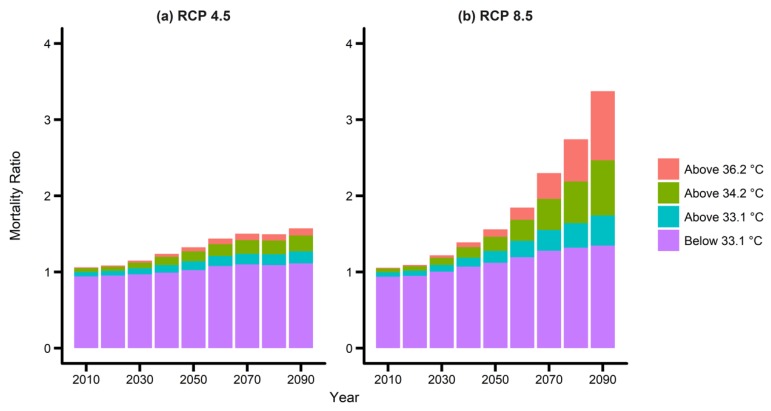
Mortality ratios according to an increased intensities and frequencies of extreme temperatures under: (**a**) RCP 4.5 and (**b**) RCP 8.5. Temperatures of 33.1 °C, 34.2 °C, and 36.2 °C correspond to the 90th, 95th, and 99th percentiles during the baseline period. RCP, Representative Concentration Pathway.

To understand the contribution of extreme temperatures (in the 90th, 95th, and 99th percentiles) to mortality, we analyzed the proportion of mortality that could be attributed to such temperatures. Figure 5 shows the MRs according to temperature changes under the RCP 4.5 and RCP 8.5. Here, 33.1 °C, 34.2 °C, and 36.2 °C are the 90-, 95-, and 99-percentile temperatures at the baseline period, and the population was assumed to remain constant at the baseline level. We found that the mortality increase was mostly due to an increased intensity and frequency of extreme temperatures. The proportion attributed to extreme temperatures was around 11% during the 2010s; this increased to 30% and 60% during the 2090s under the RCPs 4.5 and 8.5, respectively. Supplementary Figure S6 shows the increased frequencies of these extreme temperatures.

## 4. Discussion

### 4.1. Impact of Global Temperature Increases by 1.5 °C and 2 °C on Mortality

This study examined the health effects related to temperature changes under the RCP 4.5 and RCP 8.5, by using 25 GCMs, and those related to population changes under three SSP scenarios. On the basis of our mortality projection (Supplementary Table S2), the MR for the total population is below 1.17 for a temperature increase of 1.5 °C and below 1.32 for an increase of 2 °C. We found that the MR will increase by about 10% to 20% for each additional temperature increase of 0.5 °C. In particular, it will increase by 12% for a temperature increase of 2 °C, when compared to an increase of 1.5 °C under the RCP 4.5, and by 13% under the RCP 8.5. This indicates that if the global temperature increase can be kept below 1.5 °C, mortality can be decreased by about 12~13% relative to the target of 2 °C. When considering both temperature and population changes, the increase in mortality due to the temperature changes is amplified. Therefore, the benefit of limiting the temperature increase to below 1.5 °C instead of below 2 °C will be much higher because of the population change, especially under the worldwide trend of society aging.

### 4.2. Limitations

In the projection of future mortality, we assumed that the temperature–mortality relationship was constant throughout the projection period. Although people are known to acclimatize under climate change conditions, we decided not to include such an adaptation in our projection, since the rate of adaptation is uncertain. The assumption of no adaptation may have resulted in the overestimation of future mortality. In addition, we used the RCP 4.5 and RCP 8.5 when estimating future mortality under the scenario in which the temperature increases were successfully limited to below 1.5 °C and 2 °C. Under the RCP 4.5, the temperature increase saturated around 3 °C, while it did not saturate until 2100 under the RCP 8.5. This may have resulted in an overestimation of MRs, as the MRs for 1.5 °C and 2 °C under the RCP 8.5 were slightly higher than those under the RCP 4.5 (Supplementary Table S2). However, the amount of overestimation is expected to be small, given that the MRs under the RCP 4.5 and 8.5 were similar (below 5%).

## 5. Conclusions

This study examined the benefit of limiting the temperature increase below 1.5 °C or 2 °C. We projected future mortality (until the 2090s) according to temperature and population changes in seven major cities of South Korea. For the climate and population scenarios, we used RCPs and SSPs, respectively. To the best of our knowledge, this study is the first to use SSP scenarios to illustrate the impact of population changes on mortality in South Korea. Under RCP 4.5 and 8.5 scenarios, the temperature was expected to increase by 2.83 °C and 5.10 °C above pre-industrial levels, respectively, and mortality was expected to increase by 1.53 and 3.3, respectively. However, when 1.5 °C and 2 °C increase targets are met, the mortality increases will stay below 1.13 and 1.26, respectively. On the basis of our projection, limiting the global temperature increase to below 1.5 °C or 2 °C will reduce mortality by more than 35% or 21%, respectively, when compared to the mortality under RCP 4.5. In addition, we showed that mortality is expected to increase more significantly when considering the population changes. Our findings can guide the policy makers to establish policies to limit greenhouse gas emissions and to take measures to limit the global temperature increases below 1.5 °C or 2 °C. Moreover, the policy makers can establish policies for the more vulnerable elderly population to reduce the overall impact of climate change.

## Supplementary Materials

The following are available online at http://www.mdpi.com/1660-4601/15/4/822/s1; Figure S1: Overall population changes based on SSP scenarios for all, more than 60 years old and 80 years old; Figure S2: Projection of population changes based on SSP scenarios in seven major cities of South Korea; Figure S3: Mortality ratio due to only temperature change under (a) RCP 4.5 and (b) 8.5 for total population; Figure S4; Association between temperature and mortality in seven major cities of South Korea; Figure S5: Mortality ratio due to temperature and population changes under (a) RCP 4.5 and (b) 8.5 for total population; Figure S6: Portion of baseline 90-, 95-, 99-percentile temperatures (33.1℃, 34.2℃, and 36.2℃) due to temperature change; Table S1: The population of seven major cities of South Korea in 2015; Table S2: The mortality ratios (MRs) according to temperature changes only; Table S3: The mortality ratios (MRs) according to temperature and population changes.
