# Supplementary material for: Projection of Future Mortality Due to Temperature and Population Changes under Representative Concentration Pathways and Shared Socioeconomic Pathways
Source: Int J Environ Res Public Health. 2018 Apr 21;15(4):822. doi: 10.3390/ijerph15040822 (PMC5923864; doi:10.3390/ijerph15040822)
Supplement: Supplementary file 1 [file ijerph-15-00822-s001.pdf]

Projection of Future Mortality Due to Temperature and Population Changes under Representative Concentration Pathways and Shared Socioeconomic Pathways

Jae Young Lee [1], Ejin Kim [1], Woo-Seop Lee [2], Yeora Chae [3] and Ho Kim [1,*]

[1] Institute of Health and Environment and Graduate School of Public Health, Seoul National University, 1, Gwanak-ro, Gwanak-gu, Seoul 08826, Korea; jaeyoung.lee@alumni.stanford.edu (J.Y.L.); platin@snu.ac.kr (E.K.)

[2] Climate Research Department, APEC Climate Center, 12, Centum 7-ro, Haeundae-gu, Busan 48058, Korea; wslee@apcc21.org

[3] Korea Environment Institute, 370 Sicheong-daero, Sejong 30147, Korea; yrchae@kei.re.kr

* Correspondence: hokim@snu.ac.kr; Tel.: +82-2-880-2711

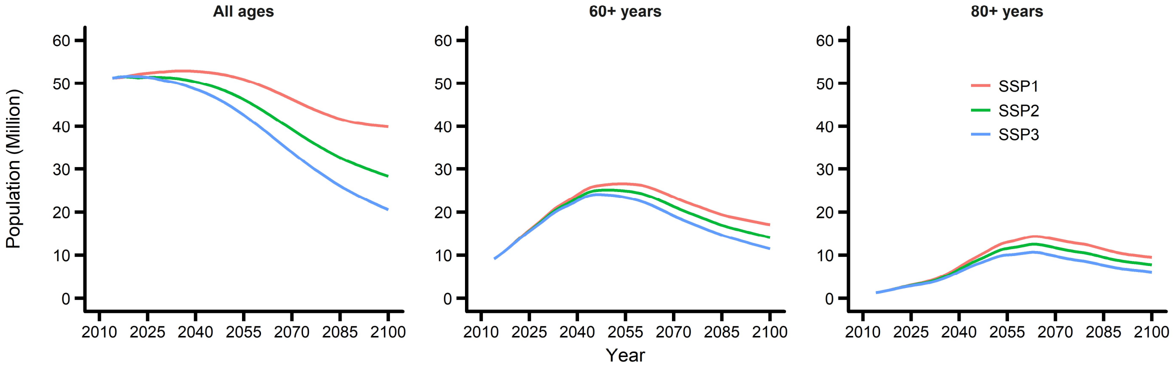

Figure S1. Overall population changes based on SSP scenarios for all, more than 60 years old and 80 years old.

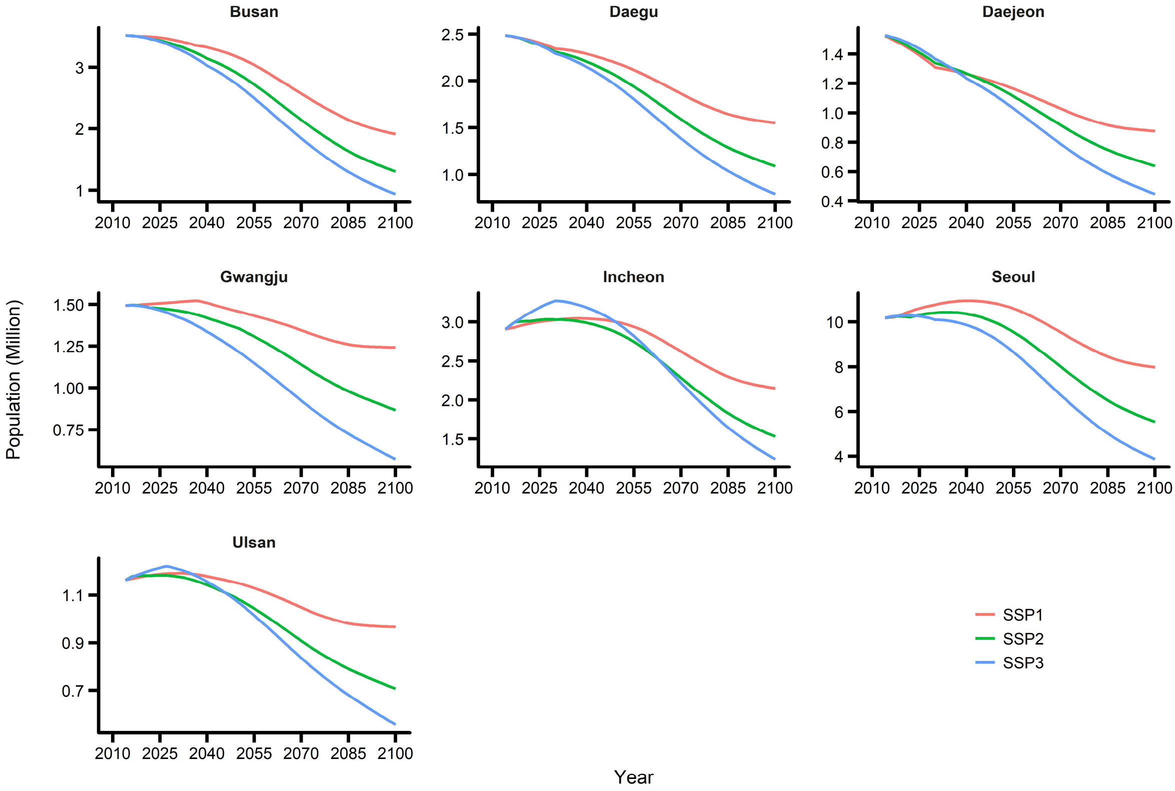

Figure S2. Projection of population changes based on SSP scenarios in seven major cities of South Korea.

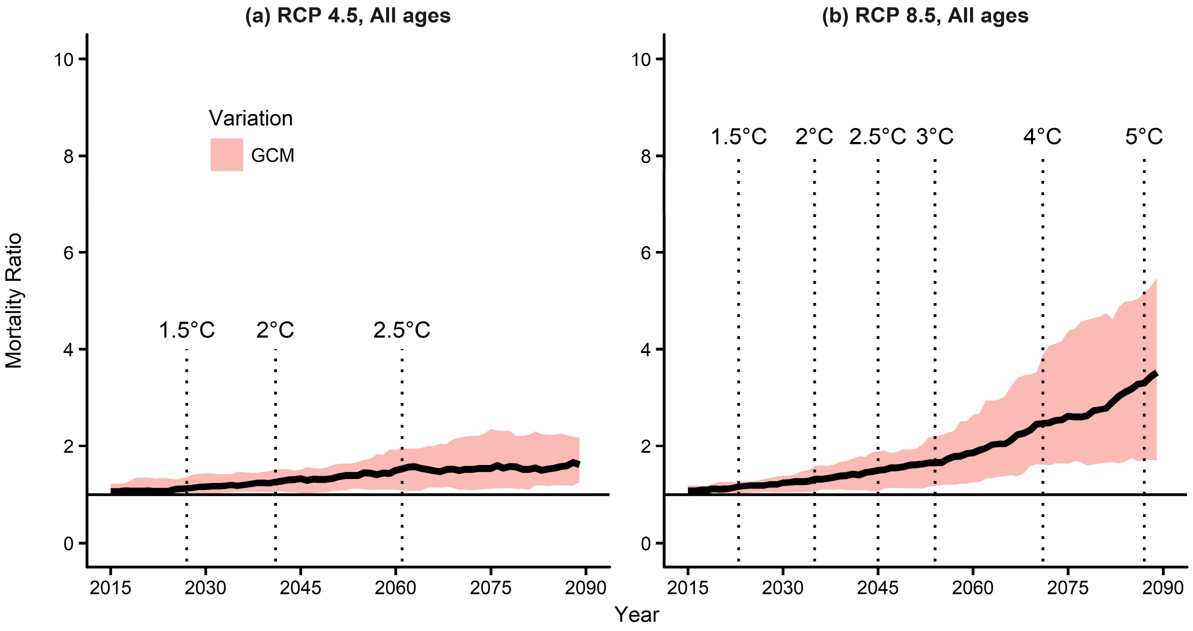

Figure S3. Mortality ratio due to only temperature change under (a) RCP 4.5 and (b) 8.5 for total population.

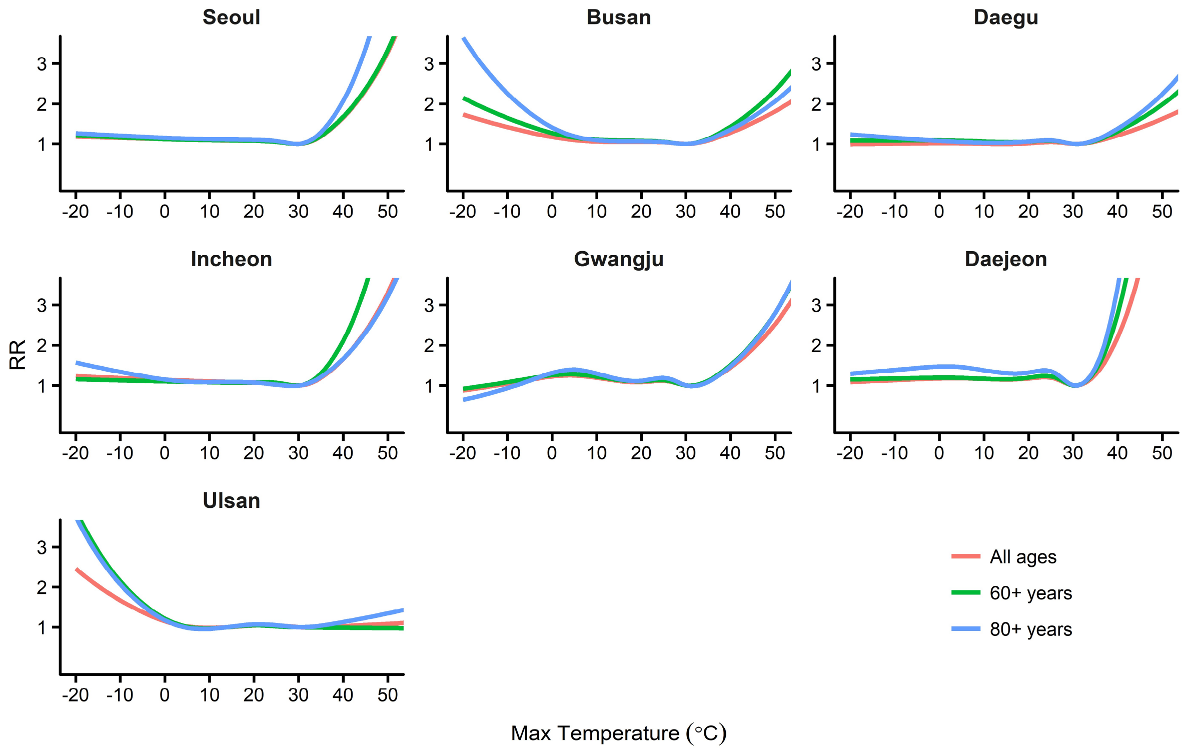

Figure S4. Association between temperature and mortality in seven major cities of South Korea.

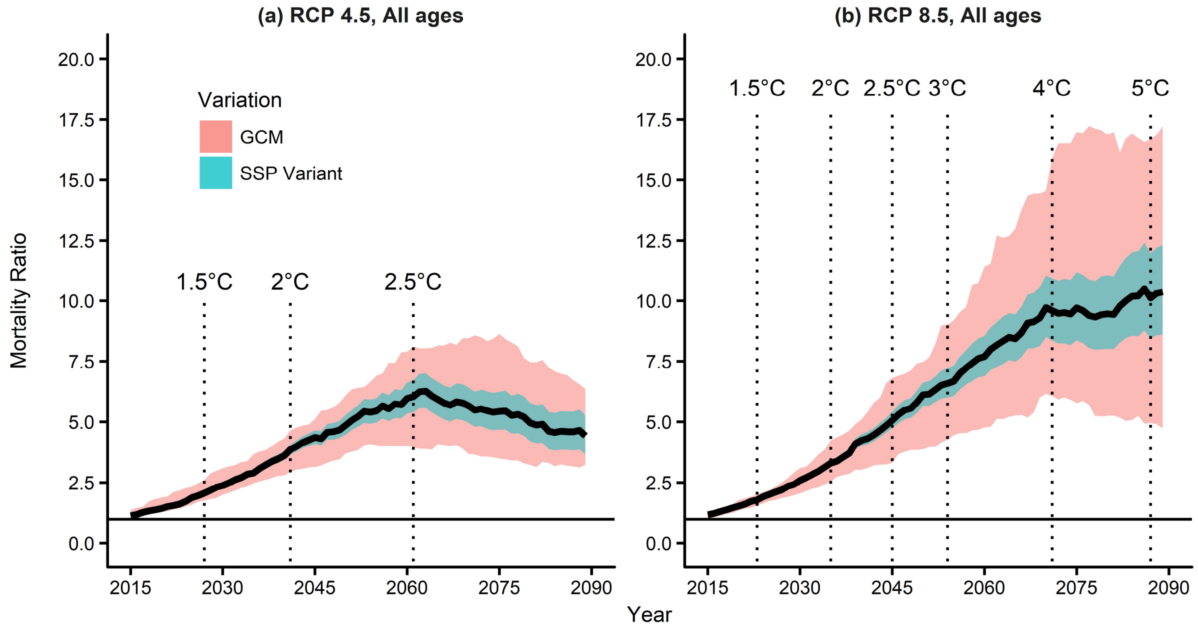

Figure S5. Mortality ratio due to temperature and population changes under (a) RCP 4.5 and (b) 8.5 for total population.

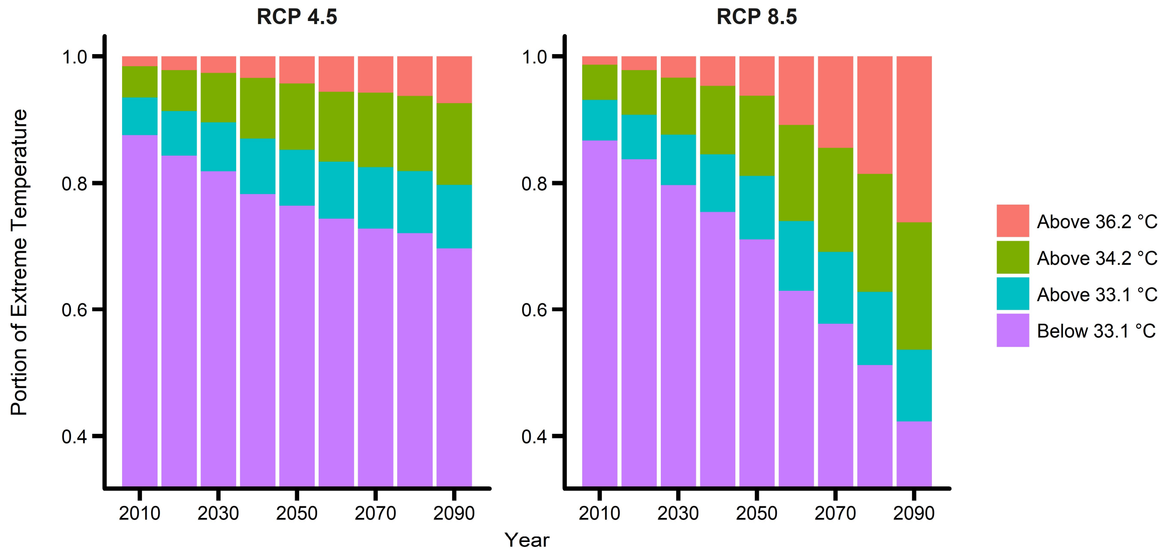

Figure S6. Portion of baseline 90-, 95-, 99-percentile temperatures (33.1℃, 34.2℃, and 36.2℃) due to temperature change.

Table S1. The population of seven major cities of South Korea in 2015.

City	Population (Millions)	Ratio
**Seoul**	10.21	19.9%
**Busan**	3.52	6.9%
**Daegu**	2.48	4.8%
**Incheon**	2.94	5.7%
**Gwangju**	1.50	2.9%
**Daejeon**	1.52	3.0%
**Ulsan**	1.17	2.3%

Table S2. The mortality ratios (MRs) according to temperature changes only.

Temperature Increase	RCP 4.5				RCP 8.5			
	**Period**	**MR (All)**	**MR (60+)**	**MR (80+)**	**Period**	**MR (All)**	**MR (60+)**	**MR (80+)**
1.5 ℃	2027 (2023-2032)	**1.13**	**1.20**	**1.12**	2023 (2019-2028)	**1.17**	**1.23**	**1.16**
2 ℃	2041 (2037-2046	**1.26**	**1.44**	**1.25**	2035 (2031-2040)	**1.32**	**1.48**	**1.34**
2.5 ℃	2061 (2057-2066)	**1.53**	**1.75**	**1.60**	2045 (2041-2050	**1.50**	**1.71**	**1.57**
3 ℃	-	-	-	-	2054 (2050-2059)	**1.65**	**1.94**	**1.77**
4 ℃	-	-	-	-	2071 (2067-2076)	**2.46**	**3.08**	**2.74**
5 ℃	-	-	-	-	2087 (2083-2092)	**3.30**	**4.23**	**3.74**

Table S3. The mortality ratios (MRs) according to temperature and population changes.

Temperature Increase	RCP 4.5				RCP 8.5			
	**Period**	**MR (All)**	**MR (60+)**	**MR (80+)**	**Period**	**MR (All)**	**MR (60+)**	**MR (80+)**
1.5 ℃	2027 (2023-2032)	**2.07**	**2.55**	**3.01**	2023 (2019-2028)	**1.78**	**2.14**	**2.45**
2 ℃	2041 (2037-2046	**3.85**	**5.04**	**7.57**	2035 (2031-2040)	**3.30**	**4.23**	**6.04**
2.5 ℃	2061 (2057-2066)	**6.04**	**8.07**	**16.35**	2045 (2041-2050	**5.06**	**6.68**	**11.61**
3 ℃	-	-	-	-	2054 (2050-2059)	**6.59**	**8.80**	**16.93**
4 ℃	-	-	-	-	2071 (2067-2076)	**9.60**	**12.93**	**26.83**
5 ℃	-	-	-	-	2087 (2083-2092)	**10.12**	**13.66**	**28.56**
